# Eruption of the Permanent Teeth

**Published:** 1888-09

**Authors:** C. N. Peirce


### ERUPTION OF THE PERMANENT TEETH.

BY C. N. PEIRCE, D. D. S.

READ BEFORE THE PENNSYLVANIA STATE SOCIETY, JUNE 6, 1888.

In writing of and tabulating the eruption of the permanent teeth, it is a matter of considerable clinical interest to recognize the early date at which these teeth begin their dentification; so that it may be recalled when teeth are met with in which calcification is very imperfectly performed. It has been shown that as early as the fifteenth week of embryonic life, preparation is made for the development of the four first permanent molars; and following close upon these, in the sixteenth week, is the inflection giving rise to the enamel-organ for the twenty anterior permanent, the successors to the twenty deciduous teeth; and from this period until the birth of the infant the germs for twenty-four of the permanent teeth are passing through their several progressive stages preparatory to receiving the salts of lime. At birth the child then has not only the twenty deciduous teeth largely advanced toward calcification, but has the germs of twenty-four permanent teeth, in twelve of which calcification commences the first year. The germs of the second permanent molars make their appearance the third month, and those of the third molars (wisdom teeth), the third year after birth.

The permanent teeth, unlike the deciduous in embryo, are constantly subjected to the influence of morbid systemic conditions during the periods of calcification, and any abnormal nutritional influence of even a few weeks' duration, if occurring during the period of coronal calcification, is sure to make an impression upon the crowns of the teeth which are at that time undergoing this process. Markings or structural defects are located at the point of calcification, and limited in extent or modified by the severity and duration of the systemic abnormality occasioning it.

If any serious nutritional disturbance occurs prior to the fifth year, the defect will be observed upon the incisors and first molar crowns, varying in location with the age and advancement of calcification. If prior to the seventh and after the third year, it will be seen on the crowns of the cuspids, bicuspids, and second molars; occurring between the eighth and twelfth years, it will probably produce some malformation of the third molars. This influence of the general health upon the teeth, inducing vices of conformation, may be assigned as a very important factor, favoring the premature loss of the third molar, development of which proceeds or is protracted through a period of childhood, when the system is liable to frequent and prolonged attacks of malnutrition, which must unavoidably interfere with perfect calcification.

While the permanent teeth in their eruption rarely produce such suffering and disastrous consequences as frequently accompany temporary dentition, there are times when the cuspids and bicuspids are so retarded in their eruption by either the persistence or the premature loss of their deciduous predecessors, or by a contracted condition of the maxillary bones, that serious trouble results. From induration of the gums or non-absorption of the anterior portion of the ramus or tuberosity the first, second, or third molar may also be the cause of much local inflammation and a febrile systemic condition ; and this is especially the case where there is an impacted third molar.

As early as the twenty-fifth week of fœtal life, the calcification of the enamel and dentine of the first molars begins ; the first year after birth the central and lateral incisors begin calcification; at four years of age the cuspids, bicuspids, and second molars begin calcification ; at eight years of age the third molars begin calcification; from the sixth to the seventh year, the four first molars are erupted; from the seventh to the eighth year, the four central incisors are erupted; from the eighth to the ninth year, the four lateral incisors are erupted; from the tenth to the eleventh year, the four first bicuspids are erupted; from the eleventh to the twelfth year, the four second bicuspids are erupted; from the twelfth to the fourteenth year, the four cuspids are erupted; from the twelfth to the sixteenth year, the four second molars are erupted; from the sixteenth to the twentieth year, the four third molars are erupted.

It will be seen that by the commencement of the sixteenth year that permanent dentition is completed, with the exception of the third molars or wisdom teeth. The variability of these is great, for while they are not unfrequently in position by the seventeenth year, they are often unerupted at the twenty-fifth, or sometimes delayed until the thirtieth or fortieth year. In this greater delay the absence of room in the arch is usually the cause, and not until some of the more anterior teeth are extracted and the walls absorbed do they make their appearance. The cuspids and second bicuspids are also less uniform in their eruption than the incisors. This may be due to either the persistence or the premature loss of their predecessors. If the deciduous cuspid be prematurely removed, the first bicuspid, which makes its appearance two years before the permanent cuspid, will move forward and take its position adjoining the lateral incisor. This necessitates the delay of the permanent cuspid some months, and when it does erupt it must encroach either on the labial or palatine surface. A similar condition results from the early loss of the second deciduous molar. The first permanent molar coming through the gums more than four years before the second bicuspid, the premature loss of the second deciduous molar would enable the first molar to occupy the space which should be protected for the bicuspid, and force the calcification of the latter to be completed beneath the crown of the first deciduous molar, or else occupy a position encroaching upon either the buccal or the lingual territory.

The associate lesions of second dentition are regarded as of trifling importance, yet not unfrequently do conditions exist at this period of the child's life which result in serious constitutional disturbance.

A want of correspondence between the growth of the root and the removal of the superimposed structures may result in stomatitis, enfeebled digestion, impaired nutrition, and fever. Wherever the terminal branches of the trifacial are distributed suffering, severe and protracted, though quite remote from the seat of the disturbance, will, until the cause is removed, baffle the best efforts of the physician. The persistence of either the inflamed and swollen or the indurated gum over the crowns of the advancing first, second or third molars, retarding their eruption and pressing the sharp edges of the calcifying roots back into the uncalcified pulps or formative papilla, cannot do less than encourage, if not produce, disorders of too serious a nature to be disregarded, and second to those of first dentition only because the increased age has lessened the child's liability to disease and increased its nutritional advantages and its resisting power. An impacted third molar at the base of the coronoid process is capable of giving as much excruciating and persistent suffering as it is possible for human nature to endure. Indeed, there is no abnormality or lesion coming in the province of the oral surgeon which demands more prompt action, or for the time more thoroughly taxes to the utmost his best judgment and skill. The removal of the anterior molar is often indicated for the purpose of giving relief; indeed, when the third molar is imbedded so that it cannot be reached, it is the only remedy.

The cause of this serious abnormality has never received the attention it deserves, and has been looked upon as a freak of nature rather than as a natural result of some potent cause.

The impaction of the third molar, so much to be dreaded in certain constitutional predispositions or idiosyncrasies, is invariably the result of one of three important factors, heredity, variation from some nervous impression, or external action or want of action, with its accumulated influence which we may term function of teeth and maxilla. The tendency of children to inherit physical peculiarities from ancestry, both near and remote, is so well established that it needs no argument to enforce it.

The influence the nervous system exerts upon the teeth and jaw is certainly well attested by their concomitant variations with the greater or less nervous energy displayed. The period of life when the brain is overtaxed or unduly stimulated, when the irritability of the nervous system is prominent in every act or movement, is a period well marked by inharmony of function and imperfect physical development.

Recognizing the fact that the trigeminus, in the fulfillment of its functions, regulates the nutrition of the tissues to which its terminal branches are distributed, we can readily appreciate the following statement made by the late Professor Anstie: "The nervous center in which the trigeminus is implanted is, of all nervous centers, the one which, in the human subject, is most liable to congenital imperfection of the kind which necessitates a break-down in its governing functions at special crises in the development of the organism." Dr. Kingsley, in his work on Oral Deformities, says: "No author on the causes of malposition of the teeth has made this direct connection between the abnormality and a disturbance of the nerve-center during the formative and eruptive period; but I find a large array of facts, confirmed by my own observations, which, in my mind, point to this conclusion only, and although other observers of similar facts have attempted, in many instances, an explanation of what they saw, they have failed to refer them to any satisfactory *primary* cause."

The influence exerted by action or want of action, use or disuse, would come under the head of "functionally produced modifications;" that these do occur every observing biologist who has written within the last century, certainly recognizes. The decrease in the size of the jaw of civilized man from that of the uncivilized or lower races is well attested, and the cause of this change in size, or modification in development, can only have resulted by or through the agency of two important factors, with their cumulative influences—decrease of function and diversion of nutrition. The modification in diet and stimulation of the brain necessarily lessens development in the one, and by diversion of nutritive current exalts the other. A change of habitat, which involves new food, different temperature, drier soil, must produce a corresponding change in the digestive, respiratory and vascular systems, and these of necessity must, in time, result in modified structural conditions.

The foregoing has a direct bearing upon the question of the retention or extraction of the sixth-year molar. We are more or less in the dark regarding the etiological factors operating to cause the loss of these teeth. There are some cases where if we could but look into the future for two or three years we could save the patient intense suffering; sometimes lasting days and even months. I speak of those cases of impaction of the third molars where necrosis results. Most notably in the lower jaw. These cases can be accounted for in that the jaws of the higher races have shortened while the size of the teeth have remained relatively the same. In such instances no alternative presents but to extract some one or more of the permanent teeth in order to prevent untoward results. Such cases are not unfrequent, and prove the exception to the rule, advanced by some, to always preserve the sixth-year molars. There are conditions of inherited tendency to disease, such as scrofula, etc., where we may predict the probable impaction of the third molar, especially if there is any history of a hereditary tendency toward impaction, where it would certainly be better to take out the sixth-year molar.

